# 3D Printability of Lysine-Modified Myofibrillar Protein Emulsions

**DOI:** 10.3390/foods14122138

**Published:** 2025-06-19

**Authors:** Lin Liao, Zilan Feng, Yoon-Yen Yow, Yajie Song, Yuxiao Liu, Lixiang Qin, Xiaofei Wu, Zhisheng Pei, Changfeng Xue

**Affiliations:** 1 School of Food Science and Engineering, Hainan Tropical Ocean University, Sanya 572022, China; 15768125399@163.com (L.L.); 18764380895@163.com (Y.S.); 17788492764@163.com (Y.L.); 18573907251@163.com (L.Q.); wuxiaofei727798@163.com (X.W.); 2 School of Food Science and Engineering, Hainan University, Haikou 570228, China; 24110832000002@hainanu.edu.cn; 3 Department of Biomedical Sciences, Sir Jeffrey Cheah Sunway Medical School, Faculty of Medical and Life Sciences, Sunway University, Sunway City 47500, Malaysia; yoonyeny@sunway.edu.my; 4 Hainan Provincial Academician Team Innovation Center, Marine Food Engineering Technology Research Center and Collaborative Innovation Center of Marine Food Deep Processing, Hainan Tropical Ocean University, Sanya 572022, China

**Keywords:** lysine, myofibrillar protein, HIPEs, 3D printability

## Abstract

This study explores the potential of lysine (Lys) and tilapia myofibrillar protein (MP) composite particles in the formulation of highly inwardly directed emulsions (HIPEs). Infrared spectroscopy, potentiometric analysis, and molecular docking studies revealed that the interaction between Lys and MP is primarily governed by hydrogen bonding and electrostatic forces. The incorporation of Lys significantly influenced the particle size, secondary and tertiary structures, solubility, and turbidity of MP. Lys-MP-stabilized HIPEs can form highly stable denser self-supporting gel network structures. Rheological analysis of HIPEs stabilized by MP showed a low energy storage modulus (G’ 110.66 Pa) and water–oil separation, therefore preventing 3D printing. However, HIPEs stabilized by Lys (especially 1.5 wt%) significantly improved the energy storage modulus (G’ 1002.10 Pa), increased viscoelasticity and thixotropic recovery, and reduced droplet size (10.84 μm), facilitating the use of HIPE inks for 3D printing. Furthermore, HIPEs stabilized with 1.5 wt% Lys-MP demonstrated superior print accuracy (91.36%), resolution, and clarity in 3D printing applications. Overall, these findings offer a promising strategy for developing Lys-MP composite particle-stabilized HIPEs tailored for advanced 3D printing technologies.

## 1. Introduction

High internal phase emulsions (HIPEs) are semi-solid emulsion gels characterized by a dispersed phase volume fraction of 74% or higher. This high internal phase leads to the formation of a gel-like network structure, resulting from the close packing and deformation of dispersed droplets [1]. Unlike conventional emulsions, HIPEs exhibit superior stability, distinctive rheological behavior, and environmentally friendly attributes, making them highly attractive for various applications, particularly in the food industry. In the context of emerging food processing technologies such as 3D printing, HIPEs are increasingly used due to their favorable mechanical properties and printability [2]. Moreover, there is a growing demand for the use of safe, naturally derived emulsifiers in place of synthetic alternatives to ensure both the functionality and safety of 3D-printed food products [1]. Examples include rice proteins [3] and β-accompanied soybean globulin proteins [4]. Among them, myofibrillar proteins (MPs) are particularly known for their widespread application in food products, attributed to their high nutritional value, abundance, and excellent digestibility [5]. MPs constitute approximately 66–77% of the total protein content in fish [6]. Due to their amphiphilic nature, fish-derived MPs exhibit emulsifying properties that contribute to improved emulsion stability [7]. In 3D food printing, the composition and rheological characteristics of the printing ink play a crucial role in determining the performance of the final printed product [8]. However, HIPEs stabilized solely with MPs are not suitable for 3D-printed food applications, as they fail to produce the desired textured appearance in the final printed product [1,9].

In recent years, studies have highlighted the functional benefits of lysine (Lys) in protein systems. Cao et al. demonstrated that alkaline Lys increased the proportion of porcine myosin monomers and improved their solubility [10]. Similarly, Li et al. [11] reported that the addition of Lys raised the pH of chicken myosin, increased the net negative charge on the myosin molecules, and promoted structural unfolding. These changes led to alterations in the protein’s secondary structure and greater exposure to hydrophobic groups. Furthermore, Lys has been shown to improve the physical stability of emulsions by modulating interfacial permeation and protein unfolding behavior, therefore increasing both the emulsion activity index and the emulsion stability index [12].

This study aims to elucidate the effects of different Lys concentrations (0–2.5 wt%) on the stability, physicochemical properties, and 3D printing performance of MP-based HIPEs. It investigates how different Lys levels influence the microstructure, rheological behavior, and overall stability of the emulsions, along with the structural characteristics and functional performance of the MP particles. The findings provide valuable insights for enhancing the use of MPs and expanding their application in 3D-printed nutritional delivery systems.

## 2. Materials and Methods

### 2.1. Materials

Tilapia fillets (160 ± 10 g) were sourced from Hainan Chengmai Xiangtai Fishery Co (Chengmai China). Lysine was obtained from Shanghai Aladdin Biochemical Technology Co., while corn oil was purchased from Yihai Kerry Golden Dragon Fish Cereals, Oils and Foodstuffs Co. (Shanghai, China). Nile blue and Nile red dyes were supplied by Macklin (Shanghai, China) and Saan Chemical Technology Co. (Shanghai, China), respectively. All other reagents used in this study were of analytical grade.

### 2.2. Preparation of the Lys-MP Solution

MP was extracted following the method described by Pei et al. [13]. A 1.5 wt% MP solution was prepared by dissolving MP in distilled water and vortexing for 1 min. Lys–MP complexes were then formed by ultrasonically mixing the MP solution with different concentrations of Lys (0 wt%, 0.5 wt%, 1.5 wt%, and 2.5 wt%) [8] for 15 min at 80 W in an ice bath (KQ-300AD, Kunshan, China). The resulting Lys–MP complexes were subsequently collected and stored at 4 °C.

### 2.3. Characterization of Lys-MP Solutions

#### 2.3.1. Sodium Dodecyl Sulphate-Polyacrylamide Gel Electrophoresis (SDS-PAGE)

Equal volumes (20 μL) of sample and loading buffer—containing Sodium Dodecyl Sulfate (SDS), Dithiothreitol (DTT), Coomassie Brilliant Blue, and buffered saline solution—were mixed thoroughly and heated in a boiling water bath for 5 min to denature the proteins. Subsequently, 10 μL of the prepared sample was loaded onto the gel for electrophoresis. The gel was run at 80 V for 20 min, followed by 120 V for 60 min. After electrophoresis, the gel was stained at 37 °C for 30 min and then decolorized until clear and distinct protein bands were observed [14]. The electrophoresis images were analyzed by image processing software ImageJ 1.8 (NIH, Bethesda, Maryland, USA).

#### 2.3.2. Solubility and Turbidity Determination of Lys-MP Solutions

Solubility measurements were conducted with modifications based on the method described by Cao et al. [10]. Samples were first diluted to a protein concentration of 25 mg/mL and then centrifuged at 10,000× *g* for 10 min at 4 °C. The protein content in the resulting supernatant was determined using the biuret method.

(1) $$Solubility=\frac{Supernatant\;protein\;concentration}{Total\;protein\;concentration}$$

Turbidity measurements were performed with modifications based on the method described by Su et al. [15]. The samples were diluted to a concentration of 25 mg/mL, and turbidity was assessed by measuring absorbance at 600 nm.

#### 2.3.3. Zeta Potential and Size Determination of Lys-MP Particles

The Zeta potential and size of Lys-MP particles were measured using a Zetasizer Pro (Malvern Panalytical, Worcestershire, UK) and a laser particle sizer (LS13 320, Beckman Coulter, CA, USA) [8]. Before measurement, the samples were diluted in pure water, placed in the measurement cell, and allowed to equilibrate for 120 s.

#### 2.3.4. Fourier Transform Infrared Spectroscopy (FTIR) Determination of Lys-MP Solutions

The secondary structure of MP was analyzed using an ATR-FTIR spectrometer (ALPHA II, Bruker, MA, USA). Lyophilized Lys-MP solutions were mixed and ground with potassium bromide (1:100 ratio) and then compressed into tablets. Spectral measurements were conducted over the range of 4000 to 400 cm^−1^ with 32 scans and a resolution of 4 cm^−1^ [6].

#### 2.3.5. Determination of Protein Tertiary Structure in Lys-MP Solutions

The tertiary structure of Lys-MP solutions was analyzed using a fluorescence spectrometer (Cary Eclipse, Agilent Technologies, Santa Clara, California, USA). Spectral measurements were recorded in the range of 300–400 nm, with an excitation wavelength set at 295 nm [15].

#### 2.3.6. Molecular Docking

The amino acid sequence of tilapia myosin (UniProt ID: I3J6T9) was retrieved from the UniProt database (http://www.uniprot.org, accessed on 18 January 2025), while the 3D structure of Lys (CID: 5962) was obtained from the PubChem database (https://pubchem.ncbi.nlm.nih.gov/, accessed on 18 January 2025). The pH setting and force field optimization were conducted using Avogadro2 v1.100.0 software, and the structures were saved in pdb format. Tilapia myosin and Lys were then pre-processed using AutoDockTools-1.5.6 software for hydrogenation, charge computation, and other necessary treatments, and saved in pdbqt format. Molecular docking was performed with AutoDock Vina 1.2.2 software, and the docking results were analyzed using Discovery Studio 3.5 Client and Open-Source PyMOL software. The protein structure was validated using SAVES 6.0.

### 2.4. Preparation of HIPEs

Lys-MP solutions (1.5 wt% MP) [8] with different Lys concentrations (0 wt%, 0.5 wt%, 1.5 wt%, and 2.5 wt%) were mixed with Φ0.80 corn oil. The HIPEs were then homogenized for 2 min at 7000 rpm using a high-speed shear mixer (XHF-DY, Scientz, Ningbo, China) equipped with a 14 mm dispersing head. The resulting HIPEs were stored at 4 °C.

### 2.5. Characterization of Lys-MP-Stabilized HIPEs

#### 2.5.1. Visual Appearance, Confocal Laser Scanning Microscopy (CLSM), and Particle Size of HIPEs

Representative images of the appearance of various MP/Lys-stabilized HIPEs were captured using a digital camera (Alpha 7II, Sony, Tokyo, Japan). The microstructure of the HIPEs was examined using confocal laser scanning microscopy (CLSM), following the method reported previously [8]. Proteins and oil phases were stained with 40 μL of Nile blue (1 mg/mL) and 50 μL of Nile red (1 mg/mL), respectively, and excited at 633 nm and 488 nm. The droplet size distribution of the HIPEs was measured using a laser particle size analyzer (LS13 320, Beckman Coulter, CA, USA).

#### 2.5.2. Rheological Characteristics of HIPE Inks

The rheological characteristics of the HIPE gels were evaluated using a rotational rheometer (MCR 92, Anton Paar, Graz, Austria) equipped with parallel plates (49.90 mm diameter, 1 mm gap) at room temperature, following the protocol outlined by Feng et al. [8]. A frequency sweep was conducted at a constant strain of 0.5% to determine the storage modulus (G’) and loss modulus (G″) across an oscillation frequency range of 0.01–10 Hz. To identify the linear viscoelastic region (LVR), a strain sweep was performed at a fixed angular frequency of 0.1 rad/s, covering a strain range of 0.1–1000%. The apparent viscosity was assessed through a flow sweep under shear rates ranging from 1 to 50 s^−1^. A three-interval thixotropy test (3ITT) was carried out to evaluate structural recovery behavior. This test consisted of three sequential shear intervals—1 s^−1^, 10 s^−1^, and again 1 s^−1^—each lasting 600 s. Recovery was quantified by calculating the ratio of the average viscosity during the third interval to that observed in the first interval [1].

### 2.6. Raman Spectroscopic Determination of HIPEs

Based on a previous research study, a laser confocal Raman spectrometer (DXR2, Thermo, Scientific, USA) was employed to analyze the Raman spectra of HIPEs stabilized with different concentrations of Lys [8]. The Raman spectroscopic analysis was conducted under the following conditions: a 532 nm laser wavelength, a 40× objective lens, an excitation power of 10 mV, an exposure time of 10 s, and a total of 20 scans.

### 2.7. Physical Stability of HIPEs

The stability of the HIPEs was evaluated under different conditions, and corresponding changes in their appearance were observed and recorded. (1) For thermal stability assessment, all HIPE samples were heated at 100 °C for 30 min, followed by rapid cooling to 25 °C. (2) For freeze–thaw stability, the samples were frozen at −20 °C for 24 h, then thawed in a 30 °C water bath for 30 min. This freeze–thaw cycle was repeated three times to evaluate structural integrity and emulsion stability under stress conditions [16]. (3) Centrifugal stability was evaluated by transferring 20 g of each HIPE sample into centrifuge tubes, followed by centrifugation at 8000 rpm for 15 min.

### 2.8. 3D Printing with HIPE Inks

HIPEs stabilized with different concentrations of Lys (0, 0.5, 1.5, and 2.5 wt%) were 3D printed using an extruder-type 3D printer (Food Bot, Hangzhou Shiyin Technology Co., Ltd., Hangzhou, China). A 15-layer “chamfered prism” model was designed with base dimensions of 20 mm, top dimensions of 25 mm, and a height of 10 mm. The printing accuracy was calculated based on the method described by Feng et al. [8]:

(2) Print Accuracy(%) = {(1 − |H_1_ − H_0_|/H_0_) + (1 − |L_a1_ − L_a_|/L_a_) + (1 − |L_b1_−L_b_|/L_b_)/3 × 100%,

Among them, H_0_, L_a_, and L_b_ respectively represent the height, upper-end length, and lower-end length of the original design shape, whereas H_1_, L_a1_, and L_b1_ are the height, upper-end length, and lower-end length after printing, respectively.

### 2.9. Statistical Analysis

Each experimental group was tested in triplicate, and the resulting data were analyzed using one-way analysis of variance (ANOVA) followed by Duncan’s multiple range test. Statistical analysis was conducted using IBM SPSS Statistics 26, with a significance threshold set at *p* < 0.05 for all comparisons.

## 3. Results

### 3.1. Characterization of Lys-MP Solutions

**Figure 1:**
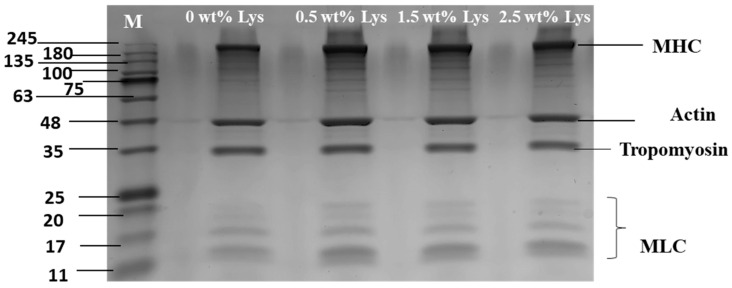
SDS-PAGE plots of MP with different Lys concentrations added.

Figure 1 illustrates the impact of different Lys concentrations on the composition of MP. The primary bands identified in the MP included the myosin heavy chain (MHC, ~200 kDa), Actin (Actin, ~48 kDa), tropomyosin (~35 kDa), and myosin light chain (MLC, ~11–20 kDa). The results of different band intensities are shown in Table 1. The bands corresponding to MHC and Actin were more intense and broader, indicating that these proteins were predominant in the MP composition. Upon treatment with Lys, the bands for MHC, Actin, and Tropomyosin became darker and broader compared to the control. This change suggests that Lys may increase the solubility of MP by increasing the pH through electrostatic interactions, leading to the observed modifications in protein band characteristics [14].

**Table 1:** The intensity of the bands of MHC, Actin, and Tropomyosin in MP with different concentrations of Lys.

Band	MHC Band Intensity	Actin Band Intensity	Tropomyosin Band Intensity
0	190,939	286,433	314,727
0.5	264,638	378,760	324,556
1.5	297,382	361,208	327,543
2.5	247,040	334,166	322,406

**Figure 2:**
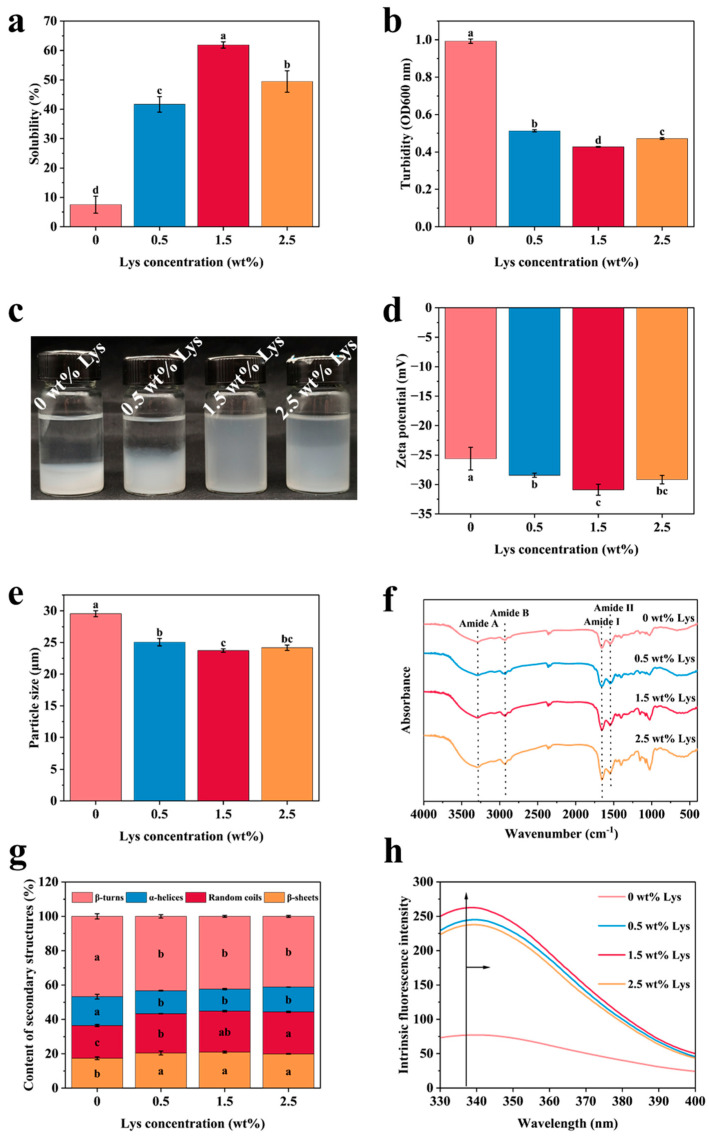
Characterization of Lys-MP: (**a**) solubility; (**b**) turbidity; (**c**) effect of different Lys treatments on the stabilization of MP aqueous solution at 4 °C for 7 days; (**d**) zeta potential; (**e**) particle size; (**f**) FTIR spectra; (**g**) content of secondary structure; (**h**) fluorescence spectroscopy of the inner courtyard. A significant difference (*p* < 0.05) is shown by different letters.

Solubility is a crucial indicator of protein denaturation and aggregation [17]. As shown in Figure 2a, MP alone showed low solubility (7.51%) in water, demonstrating its near-insolubility. However, increasing the Lys concentration significantly increased the solubility of MP, with a maximum solubility of 61.89% observed at 1.5 wt% Lys. This increase in solubility with higher Lys concentrations can be attributed to Lys’s alkaline nature, which raises the pH of the system (Table S1). The resulting electrostatic interactions between myosin molecules likely strengthen, and the charge effect disrupts the internal structure of the myosin molecules, thus improving the protein’s solubility. Cao et al. reported similar findings, noting that L-Lys increased the proportion of myosin monomers and significantly improved the solubility of oxidized porcine MP [10]. The pH of the solution decreased, intermolecular repulsion decreased, and solubility decreased upon increasing the concentration of Lys to 2.5 wt%. To investigate the emulsification mechanism of MP-based HIPEs at different Lys concentrations, it was essential to explore the interaction between Lys and MP particles. Turbidity, which reflects the aggregation of proteins within the system, displayed a trend opposite to that of solubility. As the Lys concentration increased, turbidity decreased significantly (Figure 2b). At low ionic strength, MP tended to aggregate and precipitate, while the α-helical rod-like region of myosin facilitated the formation of fibrous polymers through self-assembly [15]. Lys, being an alkaline amino acid, altered the system’s pH, which in turn influenced the electrostatic interactions and the dissociation of myosin filaments at low ionic strength. This disruption affected the protein–water interactions and the protein aggregation state. Consequently, different Lys concentrations led to changes in the solution’s pH, which impacted the electrostatic interactions, induced structural changes in MP, and ultimately affected both the solubility and turbidity of the solution.

To assess the impact of different Lys concentrations on the stability of MP, both MP alone and Lys-MP solutions were stored at 4 °C for 7 days. As illustrated in Figure 2c, the MP solution without Lys revealed rapid aggregation and precipitation, with visible phase separation occurring within just 30 min. However, the solution containing 1.5 wt% Lys remained stable throughout the 7-day storage period, maintaining a homogeneous appearance similar to its initial state. The other Lys-MP groups, however, showed visible protein aggregation and phase separation over time. These observations align with the previously reported solubility and turbidity results, further confirming the stabilizing effect of Lys at optimal concentrations.

As shown in Figure 2d, all protein samples exhibited negative surface charges. The addition of Lys significantly increased the absolute zeta potential of MP, with 1.5 wt% Lys-MP reaching the highest value of –30.89 mV. Zeta potential serves as an indicator of electrostatic interactions between particles, where higher absolute values generally correspond to improved dispersion stability and reduced protein aggregation. This suggests that Lys effectively improved the colloidal stability of MP solutions by strengthening electrostatic repulsion among protein molecules [18]. These results indicate that the addition of Lys increases the surface charge of MP particles, leading to stronger electrostatic repulsion and improved colloidal stability, with the 1.5 wt% Lys-MP solution showing the most stable behavior. As shown in Figure 2e, Lys also significantly reduced the particle size of MP compared to the untreated control, with the smallest particle size observed at 1.5 wt% Lys. This finding aligns with the results of Fan et al., who reported that Lys facilitates the dissociation of myosin and actin. The observed reduction in particle size is also consistent with the gel electrophoresis results, further supporting the role of Lys in modifying protein structure and improving dispersion stability [19].

To investigate the secondary structure, conformational changes, and molecular interactions of the protein, Fourier-transform infrared (FTIR) spectroscopy was employed to analyze the Lys-MP complexes, as shown in Figure 2f. The amide I region (1600–1700 cm^−1^), which is most sensitive to the protein backbone and widely used for secondary structure analysis, was subjected to curve fitting using an over-Gaussian function to deconvolute overlapping peaks and accurately assess structural variations [20]. The incorporation of 1.5 wt% Lys led to an evident reduction in α-helix content, accompanied by an increase in β-sheet structures and random coils compared to untreated MP, as illustrated in Figure 2g. This structural transition is attributed to the enhanced solubility induced by Lys, which promotes protein unfolding and rearrangement of secondary structures. Lys treatment facilitated the unraveling of the myosin rod tail’s α-helical regions, progressively converting them into less ordered conformations. These findings align with those of Feng et al., who reported that alkaline Lys treatment disrupts α-helical structures while increasing β-sheet formation [8]. The α-helix structure in proteins is primarily stabilized by hydrogen bonding between the carbonyl (-CO) and amino (-NH) groups along the polypeptide chain, along with contributions from electrostatic interactions. The findings indicated that the addition of Lys reduced the number of intramolecular hydrogen bonds within the MP molecule, thus increasing the exposure of hydrophobic regions. This structural disruption led to the breakdown of the α-helical conformation and its transformation into β-sheet or irregular coil structures. The resulting increase in protein–protein interactions, driven by the formation of β-sheets, facilitated the development of stable interfacial protein membranes [6]. Therefore, the addition of 1.5 wt% Lys promoted the transition of MP secondary structures toward increased β-sheet and random coil content, improving the structural flexibility, which in turn contributed to the improved stability of protein-based emulsions.

The tertiary structure of the protein was evaluated by measuring the fluorescence intensity of Lys-MP samples. As shown in Figure 2h, a gradual increase in fluorescence intensity was observed with increasing Lys concentrations, indicating that Lys addition promoted the unfolding of MP and the exposure of hydrophobic regions and tryptophan/tyrosine residues. The red-shift in the maximum emission wavelength further suggested that these residues were increasingly exposed to a more polar environment. These changes reflect a disruption of the native tertiary structure of MP, likely due to the structural loosening induced by Lys [5]. The increase in fluorescence intensity also suggested that the MP structure tended to unfold, and the spatial site-blocking of aggregate formation in a polar environment may further impede protein self-assembly and precipitation, increasing MP solubilization [15]. The 2.5 wt% Lys resulted in a decrease in fluorescence intensity, suggesting that protein re-aggregation led to the embedding of tryptophan with a decrease in fluorescence intensity.

### 3.2. Molecular Docking

**Figure 3:**
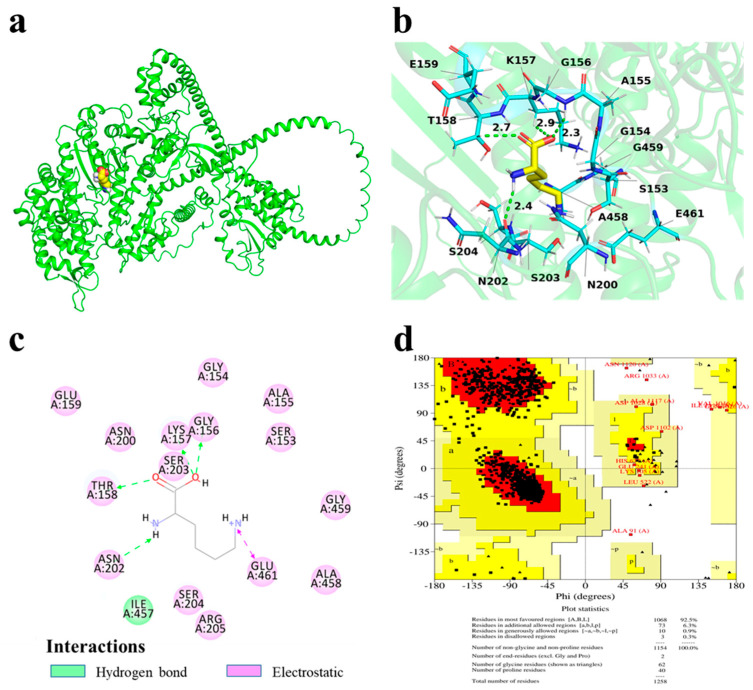
Binding patterns of Lys and MP: (**a**) three-dimensional structure of Lys complexed with MP; (**b**) three-dimensional enlargement of the binding site; (**c**) two-dimensional characterization maps of interacting residues; (**d**) Ramachandran diagram.

Molecular docking, a computational method used to elucidate molecular interactions, was employed to investigate the binding behavior between Lys and MP [21]. As shown in Figure 3, the docking model corresponding to the lowest binding energy configuration reveals that Lys entered the hydrophobic pocket of MP with a binding energy of −5.0 kcal/mol, indicating a strong and stable interaction [22]. Lys formed hydrogen bonds with the amino acid residues GLY156, ASN202, THR158, and LYS157 within the MP backbone, with the closest interaction observed at GLY156 (2.3 Å), where the strongest binding occurred. In total, four new hydrogen bonds were established. Further, electrostatic interaction was identified between Lys and GLU461 of MP. These findings are in line with the secondary structure analysis and suggest that Lys not only introduced new electrostatic interactions but also disrupted existing hydrogen bonds, leading to conformational changes in MP [8].

### 3.3. Characterization of Lys-MP-Stabilized HIPEs

#### 3.3.1. Visual Appearance, Laser Confocal Microscopy (CLSM), and Particle Size of HIPEs

**Figure 4:**
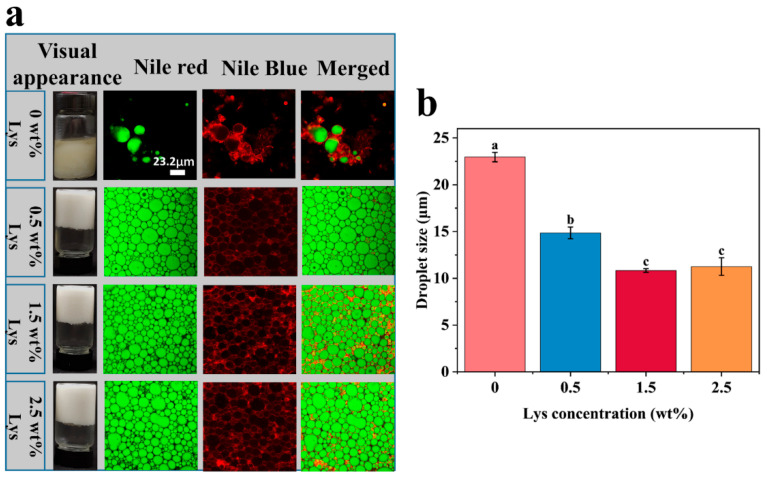
Characterization of Lys-MP-stabilized HIPEs: (**a**) CLSM images and exterior images; (**b**) droplet size. Different letters indicate statistically significant differences (*p* < 0.05).

The macroscopic appearance and microstructure of HIPEs stabilized by Lys-modified MP were examined (Figure 4a). HIPEs stabilized solely by MP revealed poor stability, characterized by evident oil–water phase separation. However, the incorporation of Lys significantly increased the emulsion’s stability, resulting in a uniform, opaque, gel-like material with a self-supporting structure that remained intact when inverted. CLSM was used to visualize the interfacial architecture of the emulsions. The images revealed irregular, polyhedral droplets with green fluorescence in the interior and red fluorescence at the droplet interface, confirming the formation of oil-in-water (O/W) type HIPEs [23]. The droplets in MP-stabilized HIPEs without Lys were large and irregular in shape. However, upon the addition of Lys, the droplet size decreased, with the smallest and most uniform droplets observed at 1.5 wt% Lys. These findings were consistent with the droplet particle size data (Figure 4b). As the Lys concentration increased, the droplets in the HIPEs became more tightly packed compared to the control group. This densification of droplets suggests that neighboring droplets share a common interfacial layer, facilitating the formation of a gel network structure. This network formation is the primary factor contributing to the semi-solid, self-supporting, gel-like properties of the HIPEs [16,23]. Secondly, the formation of irregular polygonal structures contributed to the high viscoelasticity and stability of the HIPEs. This observation aligns with previous microscopic studies that reported the formation of stable polygonal structures in HIPEs prepared from arginine and myofibrillar proteins [8].

#### 3.3.2. Rheological Characteristics of HIPE Inks

In 3D printing, the rheological properties of HIPEs significantly influence printing accuracy and structural stability. Therefore, frequency sweep, strain sweep, apparent viscosity, and 3ITT were employed to evaluate and analyze the behavior of Lys-MP-stabilized HIPEs.

**Figure 5:**
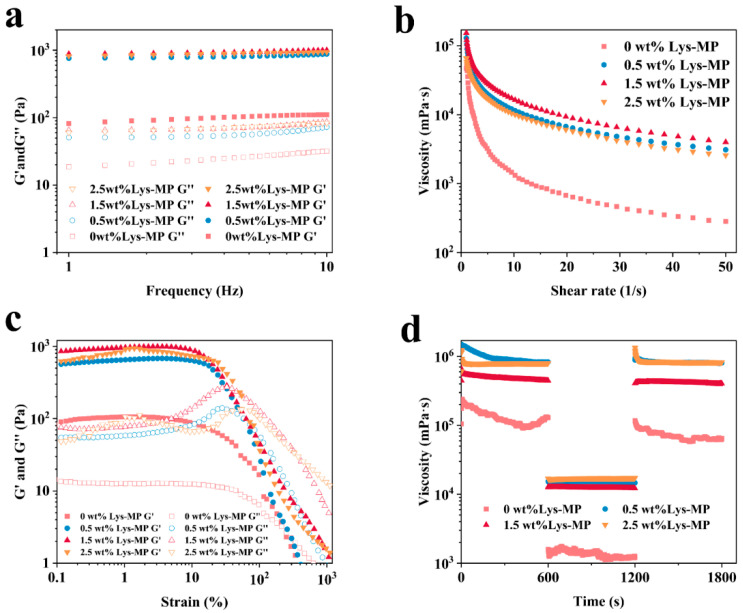
Rheological properties of HIPEs stabilized by Lys-MP: (**a**) Frequency sweep; (**b**) Strain sweep; (**c**) Shear rate sweep; (**d**) Three-interval thixotropy test (3ITT).

The frequency sweep test was used to assess the structural integrity of HIPEs, particularly their self-supporting capabilities during 3D printing. As shown in Figure 5a, the energy storage modulus (G′) was consistently higher than the loss modulus (G″) across the frequency range, indicating the formation of a stable, solid-like gel network structure. The G′ of HIPEs decreased with increasing Lys content, with the maximum structural strength observed at 1.5 wt% Lys, where the HIPEs exhibited optimal performance.

The strain sweep test was employed to evaluate the deformation resistance and stability of HIPEs during 3D printing. As shown in Figure 5b, all HIPEs displayed a linear viscoelastic region (LVR) at low strains, with no internal structural deformation. Within the LVR, G′ consistently exceeded G″, signifying that the HIPEs maintained an elastic gel state. Importantly, HIPEs stabilized with Lys showed a broader LVR and higher G′ compared to those stabilized with MP alone. The fact that G′ was an order of magnitude greater than G″ further confirms the synergistic effect of MP and Lys in forming a robust, stable network structure in HIPEs. The point of intersection, γ_co_, between G′ and G″ serves as an indicator of the stability of the HIPEs [1]. As shown in Figure 5b, γ_co_ initially increased and then decreased with increasing Lys content, indicating that an excessive amount of Lys compromised the deformation resistance and stability of the HIPEs.

A shear rate test was conducted to examine the viscosity characteristics of HIPEs under external forces (Figure 5c). The apparent viscosities of all HIPEs decreased gradually as the shear rate increased, revealing shear-thinning behavior typical of non-Newtonian (pseudo-plastic) fluids [16]. This shear-thinning property allowed the HIPEs to be easily and smoothly extruded through the 3D printing nozzle. The increase in shear rate disrupted the internal network structure of the HIPEs, leading to a reduction in the apparent viscosity of the emulsion. Droplet aggregation within the HIPEs likely contributed to variations in viscosity across different samples. As the Lys concentration increased, the number of droplets per unit volume increased, and droplet size decreased, which facilitated droplet aggregation. This aggregation, in turn, improved the apparent viscosity of the HIPEs, consistent with the microstructural findings mentioned earlier. However, the apparent viscosity of the HIPEs decreased at higher Lys concentrations (2.5 wt%), which could be attributed to the reorganization of droplets along the shear direction, disrupting the emulsion’s internal structure.

Thixotropy is commonly used as an important indicator for modeling to evaluate the recoverability of HIPEs [1]. 3ITT was employed to evaluate the structural resilience of the HIPEs. As illustrated in Figure 5d, the HIPEs showed high viscosity under low shear rates (0.1 s^−1^) during both the first and third stages, while a marked reduction in viscosity was observed in the second stage under high shear conditions. Compared to HIPEs stabilized solely with MP, those containing Lys demonstrated a recovery rate of up to 85%, indicating excellent structural recovery following the shear-induced disruption. These findings suggest that the HIPEs possess desirable shear-thinning behavior, mechanical strength, and structural stability—key attributes for 3D printing applications. To further assess their suitability, the stability of HIPEs prepared with 1.5 wt% Lys, 1.5 wt% MP, and an oil phase fraction of 0.80 will also be investigated.

### 3.4. Raman Spectroscopic Determination of HIPEs

**Figure 6:**
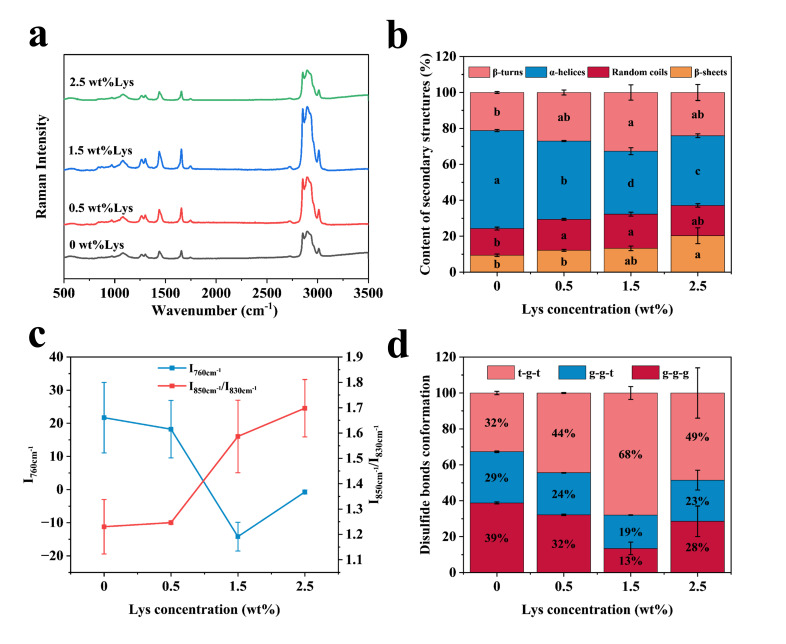
Structural characterization of protein in HIPEs: (**a**) Raman spectra in the range of 500–3500 cm^−1^; (**b**) secondary structure composition of MP based on spectral fitting; (**c**) variations in the microenvironment of tryptophan and tyrosine residues; (**d**) conformational changes in disulfide bonds. Different letters indicate statistically significant differences (*p* < 0.05).

Figure 6a presents the Raman spectra of HIPEs stabilized with different concentrations of Lys, recorded over the range of 500–3500 cm^−1^. To evaluate the secondary structure of MP within the HIPEs, Gaussian fitting was applied to the amide I region (1600–1700 cm^−1^), as shown in Figure 6b. At a Lys concentration of 1.5 wt%, a significant reduction in α-helix content was observed, accompanied by an increase in β-sheet, β-turn, and random coil structures. This shift suggests that enhanced hydrophobic interactions—driven by increased exposure to hydrophobic residues—promote protein unfolding and structural rearrangement. The decrease in α-helical structures and the corresponding rise in β-folding facilitated protein cross-linking and the formation of a gel-like network, consistent with structural transitions previously reported in chickpea protein-stabilized Pickering emulsions and striped bass myosin gels [24].

I_760cm_^−^^1^ is mainly monitored for microenvironmental changes in tryptophan residues [25]. As shown in Figure 6c, I_760cm_^−1^ displayed a decreasing trend with increasing Lys concentration, indicating progressive exposure of tryptophan residues within the MP. Moreover, the Fermi doublet intensity ratio (I_850cm_^−1^/I_830cm_^−1^) which reflects the local environment of tyrosine residues [24], remained greater than 1 across all samples. This suggests that tyrosine residues were increasingly exposed to a hydrophobic environment as Lys content rose. Overall, the increase in Lys concentration led to greater exposure of hydrophobic amino acid residues—namely tryptophan and tyrosine—therefore increasing the hydrophobicity of the MP and promoting structural rearrangements conducive to gel formation [26].

Disulfide bonds play a critical role in determining protein structure and stability. As illustrated in Figure 6d, the conformational states of disulfide bonds in MPs were analyzed using Raman spectroscopy within the 500–545 cm^−1^ range. Among the observed conformations, the t-g-t configuration (535–545 cm^−1^) showed the strongest intensity, followed by g-g-g (500–515 cm^−1^) and g-g-t (515–525 cm^−1^). Upon the addition of Lys, the disulfide bond conformation in MP progressively shifted from g-g-g to g-g-t and eventually to the more stable t-g-t form. These findings suggest that Lys not only influences protein–protein interactions but also induces conformational changes in MP at the molecular level, potentially improving gel network stability.

### 3.5. Physical Stability of HIPEs

**Figure 7:**
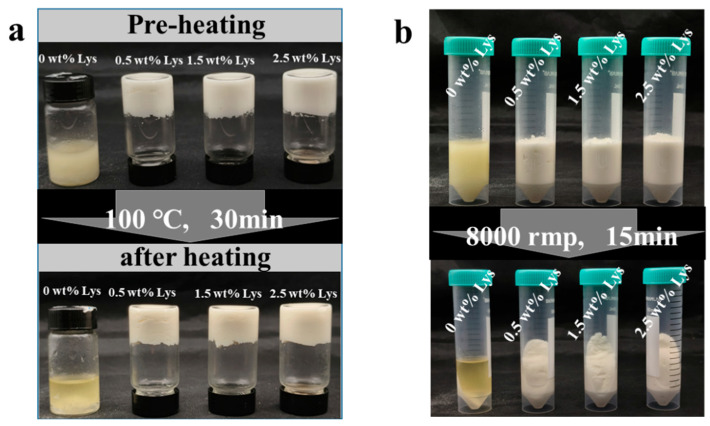

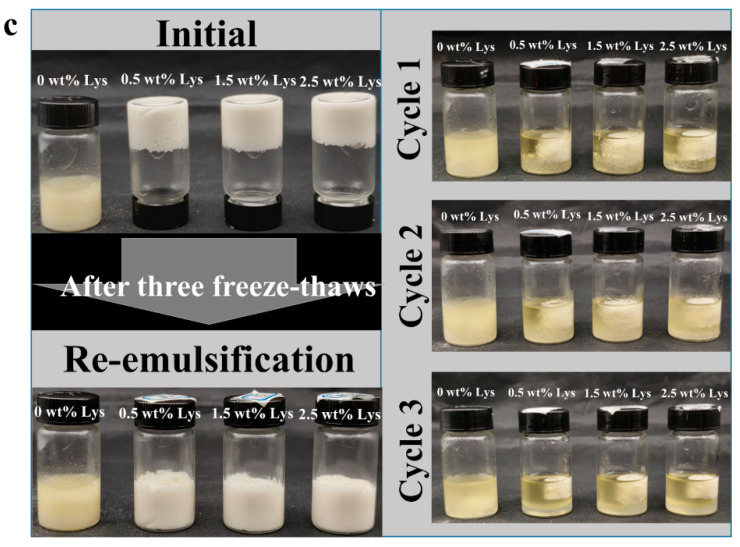
Stability assessment of HIPEs: (**a**) visual appearance of HIPEs after heat treatment at 100 °C for 30 min; (**b**) visual appearance of HIPEs following centrifugation at 8000 rpm for 15 min; (**c**) visual comparison of HIPEs before and after three freeze–thaw cycles.

Heat treatment plays a vital role in ensuring food safety during processing. To evaluate the thermal stability of HIPEs, samples were subjected to heating at 100 °C for 30 min. As illustrated in Figure 7a, HIPEs stabilized solely with MP displayed significant denaturation and aggregation following heat exposure. However, HIPEs formulated with the addition of Lys retained a consistent appearance before and after heating, indicating that Lys contributes to maintaining the integrity of the gel network and increasing thermal stability. These findings are consistent with previous studies showing that emulsions stabilized with perilla protein and chitosan exhibit improved resistance to thermal degradation [23]. The results demonstrated that Lys-stabilized HIPEs possess excellent thermal stability, making them well-suited for application in thermally processed food systems.

HIPE-based inks are widely employed in 3D-printed food products, where frozen storage is often employed to extend shelf life. Therefore, evaluating the freeze–thaw stability of HIPEs is essential. As shown in Figure 7b, all HIPE formulations displayed a visible emulsion rupture after undergoing three freeze–thaw cycles. This behavior is consistent with previous observations in HIPEs stabilized by ovalbumin [27] and soybean protein isolate [16]. The rupture is primarily attributed to structural damage caused by ice crystals formed during freezing, which compromise the emulsion network upon thawing [28]. Furthermore, following three freeze–thaw cycles, HIPEs stabilized by Lys could re-emulsify, but HIPEs stabilized by MP remained separated into water and oil.

The centrifugation results are presented in Figure 7c. Upon high-speed centrifugation, HIPEs stabilized solely by MP revealed clear phase separation into three distinct layers: an oil layer, an emulsified layer, and a water layer. However, HIPEs stabilized with Lys showed only two layers—an emulsified layer and a water layer. HIPEs containing 1.5 wt% Lys showed the least amount of water separation, suggesting superior resistance to centrifugal force. This increased stability is likely attributed to a more robust gel network structure and the presence of smaller droplet sizes [29].

### 3.6. HIPE Inks for 3D Printing

**Figure 8:**
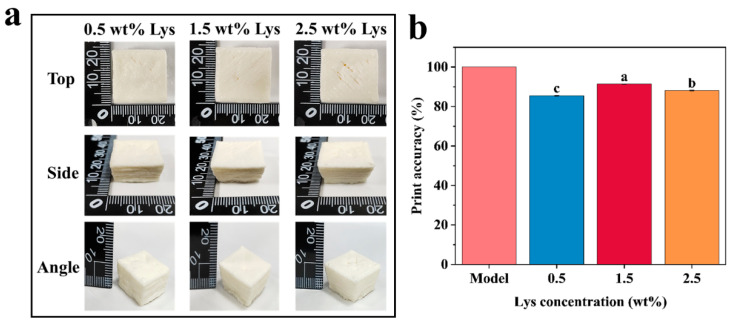
(**a**) Photographs of 3D models printed with different Lys concentrations of stabilized HIPEs. (**b**) Print accuracy. Different letters indicate statistically significant differences (*p* < 0.05).

HIPEs stabilized solely by MP exhibited poor structural stability and lacked self-supporting capacity, rendering them unsuitable for 3D printing. As shown in Figure 8, the incorporation of Lys significantly improved the extrudability, injectability, and printing precision of HIPEs [23]. HIPEs stabilized with 1.5 wt% Lys and MP successfully printed into “chamfered prism” structures with well-defined shapes, smooth surfaces, and high resolution. The improved printability is likely due to the formation of a more rigid gel network structure through cross-linking between Lys and MP, which improved the ink’s elasticity, thixotropy, and self-supporting characteristics. Furthermore, no nozzle clogging was observed during the printing process [30]. These findings underscore the beneficial role of Lys in improving the structural performance of MP-stabilized HIPEs and demonstrate their strong potential for application in 3D food printing.

## 4. Conclusions

In this study, Lys-MP composite particles were successfully developed to stabilize high internal phase emulsions (HIPEs) suitable for 3D printing applications. Lys, mediated through hydrogen bonding and electrostatic interactions, induced conformational shifts in MP with a significant decrease in particle size (1.5 wt% Lys-MP minimum), increased absolute value of the zeta potential, and secondary structure rearrangement (decrease in the α-helix content and increase in the β-folding and irregularly coiled content), facilitating protein unfolding with exposure of hydrophobic moieties. MP-stabilized HIPEs alone showed water–oil separation and could not be printed, but HIPEs stabilized by adding Lys displayed good printability and were highly stable. The 1.5 wt% Lys-MP-stabilized HIPEs formed a dense self-supporting gel network structure, with CLSM displaying smaller homogeneous polyhedral droplets and a droplet size of 10.84 μm. These emulsions showed significantly optimized rheological properties and improved stability in heat treatment, centrifugation, and freeze–thaw cycles. HIPEs stabilized with 1.5 wt% Lys-MP successfully printed well-defined ‘chamfered prism’ structures with improved precision, resolution, and shape fidelity. These findings provide valuable insights into the structural design of myofibrillar protein-based emulsions and highlight their potential in advanced food fabrication and nutrient delivery systems through 3D printing.

## Data Availability

The original contributions presented in the study are included in the article, further inquiries can be directed to the corresponding author.

## Supplementary Materials

The following supporting information can be downloaded at: https://www.mdpi.com/article/10.3390/foods14122138/s1.

## Abbreviations

The following abbreviations are used in this manuscript:

Lys	Lysine
MP	myofibrillar protein
HIPEs	highly inwardly directed emulsions
SDS-PAGE	Sodium dodecyl sulphate-polyacrylamide gel electrophoresis
MHC	myosin heavy chain
FTIR	Fourier transform infrared spectroscopy
CLSM	Confocal laser scanning microscopy
