# Supplementary material for: 3D Printability of Lysine-Modified Myofibrillar Protein Emulsions
Source: Foods. 2025 Jun 19;14(12):2138. doi: 10.3390/foods14122138 (PMC12191990; doi:10.3390/foods14122138)
Supplement: Supplementary file 1 [file foods-14-02138-s001.zip › foods-3659350-supplementary.pdf]

Supplementary Material

3D printability of Lysine-modified myofibrillar protein emulsions

Lin Liao[1], Zilan Feng[2], Yoon-Yen Yow[3], Yajie Song[1], Yuxiao Liu[1], Lixiang Qin[1], Xiaofei Wu[1], Zhisheng Pei[1, 4, *], Changfeng Xue[1, 4,*]

1 School of Food Science and Engineering, Hainan Tropical Ocean University, Sanya 572022, China; 15768125399@163.com (L.L.); 18764380895@163.com (Y.S.); 17788492764@163.com (Y.L.); 18573907251@163.com (L.Q.); wuxiaofei727798@163.com (X.W.)

2 School of Food Science and Engineering, Hainan University, Haikou 570228, China; 24110832000002@hainanu.edu.cn

3 Department of Biomedical Sciences, Sir Jeffrey Cheah Sunway Medical School, Faculty of Medical and Life Sciences, Sunway University, Sunway City 47500, Malaysia; yoonyeny@sunway.edu.my

4 Hainan Provincial Academician Team Innovation Center, Marine Food Engineering 6 Technology Research Center and Collaborative Innovation Center of Marine Food 7 Deep Processing, Hainan Tropical Ocean University, Sanya 572022, China

* Correspondence: peizhis@hntou.edu.cn (Z.P.); xuecf@hntou.edu.cn (C.X.)

Supplementary experimental methods

1. Determination of the pH of Lys-MP solutions

Lys-MP solutions (MP concentration of 1.5 wt% and Lys concentrations of 0, 0.5, 1.5 and 2.5 wt%) were prepared using a pH meter (PB-10, Sartoriu, Göttingen, Germany).

Table S1. Lys-MP solutions with different Lys concentrations of pH value.

Lys concentration (wt%)	pH
0	7.28±0..02[d]
0.5	10.00±0.02[c]
1.5	10.32±0.02[a]
2.5	10.12±0.01[b]

Note: Data are expressed as the mean ± SD (n = 3). The different letters in every row indicate significant difference at $p< 0.05$.
